# Time-in-Range With Insulin Versus Metformin in Gestational Diabetes Mellitus Using Continuous Glucose Monitoring: A Randomized Control Study at a Tertiary Care Centre in South India

**DOI:** 10.7759/cureus.61849

**Published:** 2024-06-06

**Authors:** Abhishek Pandey, Balaji Tejerao Naik, Rajini Uday, Channabasappa Shivaprasad

**Affiliations:** 1 Medicine, Institute of Medical Sciences, Banaras Hindu University, Varanasi, IND; 2 Endocrinology, Sapthagiri Institute of Medical Sciences & Research Centre, Bangalore, IND; 3 Obstetrics and Gynaecology, Sapthagiri Institute of Medical Sciences & Research Centre, Bangalore, IND

**Keywords:** continuous glucose monitoring (cgm), gestational diabetes mellitus, hyperglycemia, metformin, time in range (tir)

## Abstract

Background

The prevalence of gestational diabetes mellitus (GDM) is increasing globally. When diet and lifestyle modifications are inadequate for control, managing GDM often involves insulin or metformin. Metformin's oral administration option improves patient compliance and acceptance, but concerns about its use persist, necessitating careful evaluation. Comparative studies between insulin and metformin in GDM are scarce. In pregnancies complicated by diabetes, precise glucose control is crucial for maternal-fetal well-being, and continuous glucose monitoring (CGM) plays a valuable role in achieving recommended targets. CGM provides comprehensive glucose profiles, including postprandial glucose excursions and details about time spent in hypoglycemia, euglycemia, and hyperglycemia. The time-in-range (TIR) metric, when used alongside A1C, offers more actionable information than A1C alone. To the best of our knowledge, no published trials compare TIR in GDM with metformin or insulin aspart/detemir, specifically focusing on CGM metrics. This randomized controlled trial (RCT) aims to assess TIR in women with GDM treated with either metformin or insulin.

Materials and methods

This study was a non-inferiority randomized control trial evaluating TIR in GDM using continuous glucose monitoring with metformin or insulin. Forty-four women with GDM were enrolled. The diagnosis of GDM was based on the International Association of Diabetes and Pregnancy Study Groups (IADPSG) criteria. CGM readings were collected for 14 days after sensor activation.

Results

In our study, 44 women with GDM completed the protocol, with 22 in the Metformin group and 22 in the Insulin group. Baseline characteristics did not differ between the groups. Age, BMI pre-gravid, BMI at 28 weeks, parity, family history of diabetes mellitus, previous history of GDM, glycated hemoglobin (HbA1c), oral glucose tolerance tests (OGTT) at zero hours, one hour, and two hours, as well as gestational weeks, did not significantly differ between the two groups. The metformin and insulin groups did not differ significantly in CGM metrics, including TIR, time above range, time below range, mean glucose, and glucose management indicator.

Conclusion

Based on our findings, the metformin and insulin groups did not differ in CGM metrics, including TIR, time above range, time below range, mean glucose, and glucose management indicator. In clinical practice, CGM metrics complement fasting blood glucose, postprandial glucose, and HbA1c as appropriate and useful clinical targets and outcome measurements. Metformin's oral administration option offers advantages such as improved patient compliance and acceptance in women with GDM.

## Introduction

Gestational diabetes mellitus (GDM) is defined as hyperglycemia diagnosed for the first time during pregnancy, which is not overt diabetes [1]. The prevalence of GDM is on the rise worldwide. In cases where diet and lifestyle modifications are insufficient for control, GDM management often involves the use of insulin or metformin. Metformin offers advantages such as improved patient compliance and acceptance due to its oral administration option. However, concerns about metformin use in GDM persist, necessitating a careful evaluation of its justification. The Metformin in Gestational Diabetes (MiG) study demonstrated higher acceptability for metformin over insulin, despite metformin's gastrointestinal side effects [2]. Some studies have raised caution regarding metformin use in GDM, citing an increased risk of preterm birth [2,3]. Comparative studies between insulin and metformin in GDM are limited.

In pregnancies complicated by diabetes, where precise glucose control is crucial for maternal-fetal well-being, continuous glucose monitoring (CGM) plays a valuable role in managing and achieving recommended targets [4,5]. CGM provides comprehensive glucose profiles, including postprandial glucose excursions and details about the average time spent in hypoglycemia, euglycemia, and hyperglycemia [4,5]. The time-in-range (TIR) metric, when used alongside A1C, offers more actionable information than A1C alone. It's important to note that glycated hemoglobin (HbA1c) test is not typically used in pregnancy [4,5].

To the best of our knowledge, there are no published trials comparing TIR in GDM with metformin or insulin aspart/detemir, specifically focusing on CGM metrics. Therefore, we designed a randomized controlled trial (RCT) to assess TIR in women with GDM treated with either metformin or insulin.

## Materials and methods

Study design, subjects, and eligibility criteria

This was a non-inferiority randomized control trial to evaluate TIR in GDM with the use of metformin or insulin using CGM. This study enrolled 44 women with GDM from the Department of Endocrinology and the Department of Obstetrics and Gynaecology, Sapthagiri Institute of Medical Sciences, Bangalore, India, between September 25, 2023, and February 29, 2024. The study was registered under the Clinical Trials Registry - India (ICMR-NIMS) (registration number: CTRI/2023/09/057976). The study protocol was approved by the Institutional Ethics Committee of Sapthagiri Institute of Medical Sciences and Research Centre (approval number: SIMS&RC/EC/14/2023), and written informed consent was obtained from each subject prior to the study. This study was performed in accordance with the Declaration of Helsinki.

The diagnosis of GDM was based on the International Association of Diabetes and Pregnancy Study Groups (IADPSG) criteria [6], where the patient was diagnosed with GDM if any one of the following cut-offs in the 75g oral anhydrous glucose tolerance test was fulfilled: fasting plasma glucose ≥ 92 mg/dl, one-hour glucose 180 mg/dl, and/or two-hour glucose ≥153 mg/dl). Our center practices universal screening for GDM during the first encounter with IADPSG criteria. If an early screen was negative, then women are re-screened at 24-28 weeks of gestation.

The inclusion and exclusion criteria are given in Table 1. 

**Table 1:** Inclusion and exclusion criteria

Inclusion criteria	Exclusion criteria
Gestation age 12-30 weeks	Pre-gestational Type 1 or Type 2 diabetes mellitus
Singleton pregnancy	Newly diagnosed overt diabetes in pregnancy [HbA1c ≥ (6.5%), fasting glucose ≥ 126 mg/dl, random glucose ≥ 200 mg/dl]
Confirmed GDM (75g oral glucose tolerance test: fasting plasma glucose ≥ 92 mg/dl, 1-hr glucose 180 mg/dl, and/or 2-h glucose ≥153 mg/dl) IADPSG criteria	Endocrine conditions leading to Hyperglycemia
	Known chronic infections

Sample size

A study by Rasmussen et al. reported that the difference observed in the TIR levels between the two groups was 4.5% [7]. The upper limit of the confidence interval, 10%, was considered a non-inferiority margin for the current study. With 80% power and a 5% level of significance, the number of patients required in each arm was 18. After accounting for a dropout of 20% the number of patients required in each arm was decided as 22 subjects, a total of 44 for the study.

Study protocol

**Figure 1:**
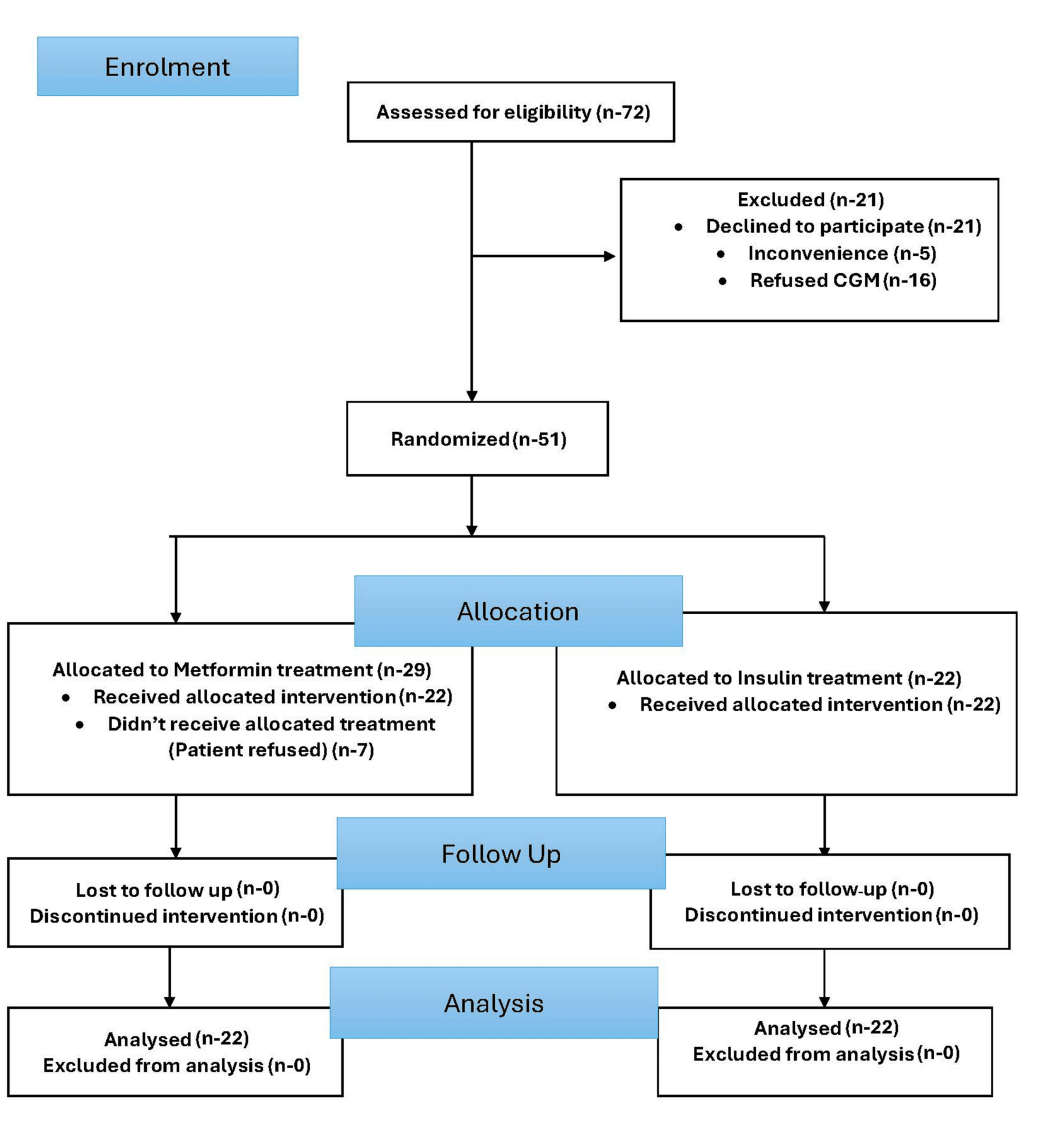
CONSORT flow diagram CONSORT: Consolidated Standards of Reporting Trials; CGM: continuous glucose monitoring

Random allocation software was used to perform randomization into two groups: the Metformin group and the Insulin group. A total of 72 women with GDM were assessed for eligibility. Of these, 21 women declined to participate: five gave a reason of inconvenience and 16 refused CGM devices. So, the remaining 51 women with GDM were randomized into two groups: 29 were allocated to the metformin group and 22 were allocated to the insulin group. Out of 29 participants in the metformin group, 22 received allocated intervention but seven patients refused to receive interventions. Out of 22 participants who were allocated to insulin treatment, all received the intervention. So, in the final analysis, there were 22 women with GDM in the metformin group as well as in the insulin group. The CONSORT flow diagram is shown in Figure 1.

Baseline clinical characteristics were obtained for two groups. Both metformin and insulin groups were provided equal care and education. The CGM device that was used in our study was FreeStyle Libre Pro (Abbott Laboratories, Chicago, Illinois, United States), which is inserted just under the skin to record the glucose level every 15 minutes. The software provided by the manufacturer for capturing and analysis of CGM readings was used. FreeStyle Libre Pro can provide CGM readings till 14 days after activation of its sensor.

Metformin tablet was started at a dose of 500 mg once or twice daily with food and increased over a week to a maximum dosage of 2000 mg based on the clinician's discretion. Insulin used in the study was aspart insulin as a bolus while detemir was used as basal insulin. In the insulin group, basal or basal plus or basal-bolus insulin regimen and dosage were decided as per the clinician's discretion, which was based on standard clinical practice. 

The participants were instructed to measure self-monitoring blood glucose as per standard clinical practice, the dosage of metformin or insulin was modified as per self-monitoring blood glucose readings. Capillary blood glucose target aimed at fasting and preprandial blood glucose concentration < 95 mg/dL, one-hour postprandial blood glucose concentration < 140 mg/dL, and two-hour postprandial glucose concentration < 120 mg/dL. Participants were instructed to record meals, hypoglycemic events, dosage of insulin or metformin, and exercise in a standardized log sheet. As per study protocol, we reviewed women with GDM who were on a CGM device every week for two weeks and then at 28 weeks of gestation.

Definition of CGM metrics

TIR

As per guidance on target for glycemic control during pregnancy, TIR should be > 70% of the TIR of 63-140 mg/dL [4,5]. This metric requires attention when interpreting CGM data and the ambulatory glucose profile (AGP) reports on which they are summarized, as default settings for nonpregnancy ranges need to be adjusted for pregnancy.

Time Above Range (TAR)

As per guidance on target for glycemic control during pregnancy, TAR target in pregnancy is <25% of the time with >140 mg/dL [4,5].

Time Below Range (TBR)

As per the guideline on target for glycemic control during pregnancy, TBR target in pregnancy is < 4% of the time with 54-62 mg/dL [4,5].

Glucose Management Indicator (GMI)

GMI is calculated from average CGM glucose. GMI gives A1C levels that would usually expected from a large number of individuals with diabetes who have the same average CGM glucose level. Laboratory A1C might be similar to, higher than, or lower than GMI [8,9]. As per guidance on target for glycemic control during pregnancy, ADA recommends GMI<6 [4,5].

Statistical analysis

Descriptive statistics were reported as mean with standard deviation and number with percentages. The assumption of normality was assessed using a Q-Q plot. A comparison of clinical parameters between metformin and insulin was performed using an independent t-test. P-value less than 5% was considered statistically significant. For the primary outcome variable, TIR, 95% confidence interval (CI) was estimated, and the fixed non-inferior margin was checked against 95% CI. All the analysis was performed using IBM SPSS Statistics for Windows, Version 25.0 (Released 2017; IBM Corp., Armonk, New York, United States).

## Results

In our study, a total of 44 women with GDM completed the protocol with 22 randomized to the metformin group and 22 to the insulin group. Baseline characteristics did not differ between the two groups. Age in years, BMI pre-gravid, BMI 28 weeks, parity, family history of diabetes mellitus, previous history of GDM, HbA1c, oral glucose tolerance tests (OGTT) at zero hours, one hour, and two hours, and gestation weeks did not significantly differ between the two groups. Baseline characteristics between metformin and Insulin groups are shown in Table 2.

**Table 2:** Comparison of clinical characteristics between metformin and insulin groups DM: diabetes mellitus; OGTT: oral glucose tolerance test

Characteristics	Metformin (N=22)	Insulin (N=22)	p-value
Age (years), mean ± SD	31.5 ±5.08	31.3 ± 4.82	0.880
BMI pre gravid (kg/m^2^), mean ± SD	27.1 ± 1.81	27.2 ± 1.77	0.887
BMI 28 weeks (kg/m^2^), mean ± SD	29.9 ± 1.81	29.5 ± 1.76	0.414
Parity, n (%)	7 (31.8)	8 (36.4)	0.750
Family history of DM, n (%)	12 (54.5)	14 (63.6)	0.540
Previous history of DM, n (%)	4 (18.2)	5 (22.7)	0.709
OGTT(mg/dl) at zero hours, mean ± SD	99.4 ± 6.87	100.4 ± 5.80	0.605
OGTT(mg/dl) at one hour, mean ± SD	178.6 ±9.25	179.1 ± 7.94	0.848
OGTT(mg/dl) at two hours, mean ± SD	152.5 ± 6.71	153.0 ± 7.09	0.812
Gestation (weeks), mean ± SD	22.7 ± 3.17	22.8 ± 2.57	0.946
HbA1c (%), mean ± SD	5.58 ± 0.35	5.62±0.28	0.675
Time in range (%), mean ± SD	69.9 ± 7.94	70.1 ± 7.34	0.953
Mean glucose (mg/dl), mean ± SD	125.0 ± 10.3	124.1 ± 10.3	0.771
Glucose management indicator (%), mean ± SD	6.30 ± 0.24	6.27 ± 0.25	0.771
Time above target (%), mean ± SD	16.7 ± 6.05	15.7 ± 4.99	0.536
Time below target (%), mean ± SD	13.3 ± 3.18	14.2 ± 4.05	0.413

TIR

The metformin group and the insulin group did not differ significantly in TIR. The metformin group's TIR (%) was 69.9± 7.94 and the insulin group's TIR (%) was 70.1±7.34. TIR was the primary outcome variable for which the study was powered. The non-inferiority margin set in our study for TIR comparison between two groups was 10%. The result showed non-inferiority of metformin in comparison to Insulin in treating GDM.

TAR and TBR

The metformin and insulin groups did not show a significant difference in TAR. The metformin group's TAR(%) was 16.7±6.05 while the insulin group's TAR (%) was 15.7±4.99. Similarly, the metformin and insulin groups did not show a significant difference in TBR. The metformin group's TBR (%) was 13.3±3.18 while the insulin group's TBR (%) was 14.2±4.05.

GMI

In our study, GMI in the metformin group was 6.30±0.24 while in the insulin group, it was 6.27±0.25, which showed no significant difference.

**Figure 2:**
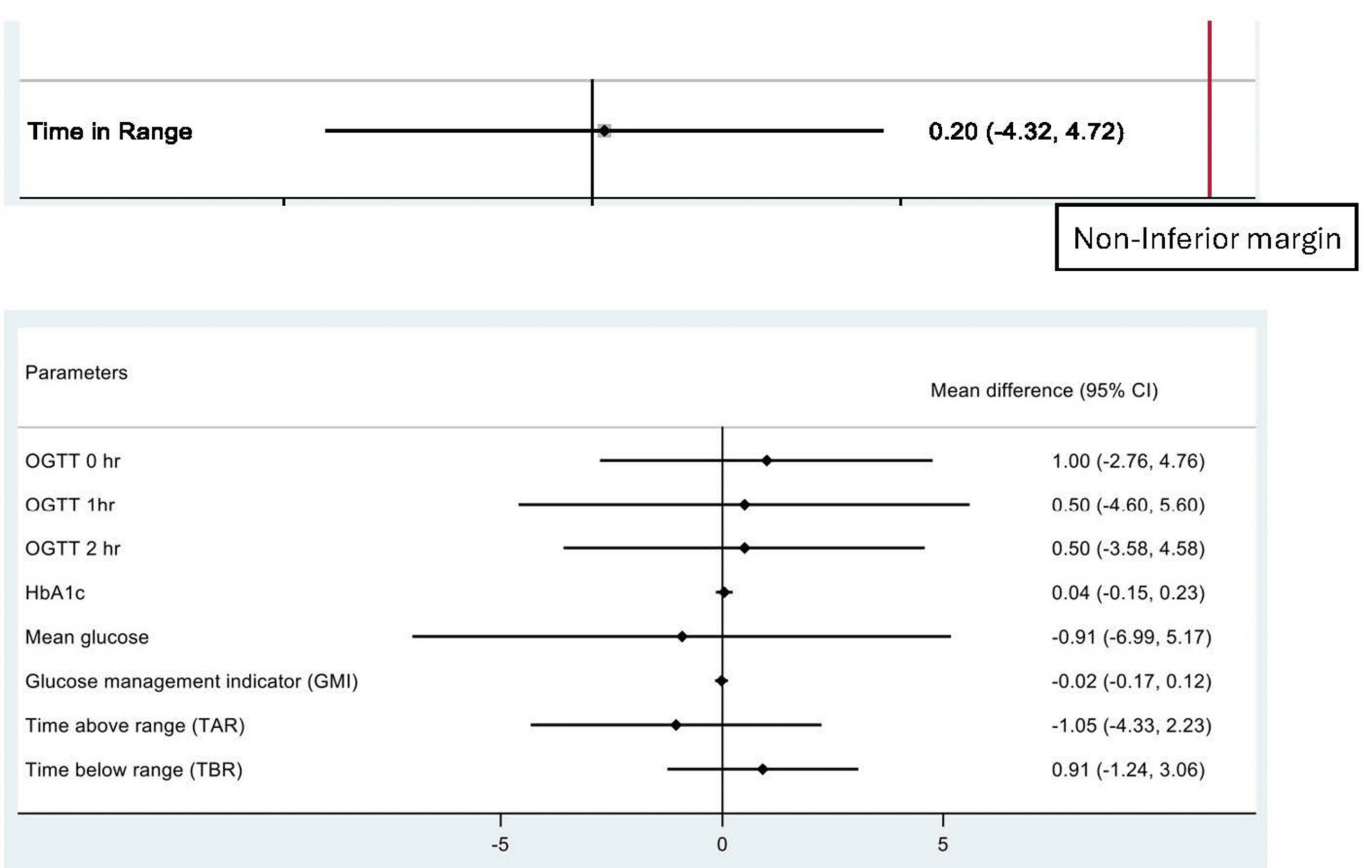
Plots showing non-inferior results OGTT: oral glucose tolerance test; HbA1c: glycated hemoglobin

The plots showing non-inferior results are given in Figure 2. 

## Discussion

Our results suggest that the TIR metric calculated by CGM devices in patients with GDM who were on metformin or insulin was statistically similar. To our knowledge, similar findings have not been shown before. A meta-analysis on metformin vs insulin in the management of GDM by Feng et al. concluded that metformin was comparable to insulin in glycemic control and neonatal outcome [10]. Glycemic control parameters taken in previous studies were fasting blood glucose, postprandial glucose levels, and HbA1c. In our study, we compared different CGM metrics in women with GDM in both metformin and insulin arms. In clinical practice, CGM metrics are both appropriate and useful as clinical targets and outcome measurements that complement fasting blood glucose, postprandial glucose and HbA1c. The use of CGM continues to expand in clinical practice. Our data is reassuring for clinicians who use metformin or insulin in GDM.

The other CGM metrics like TAR, TBR, and GMI are also statistically similar in the metformin or insulin groups. This showed the non-inferiority of using metformin in comparison to insulin among women with GDM in terms of various CGM metrics not only in TIR for which study was primarily powered.

In our study, baseline characteristics in the metformin and insulin groups were similar. The main strength of this study is that it was a randomized trial. There are very limited RCTs using CGM in GDM. Paramasivam et al. used CGM in 50 women requiring insulin for GDM [7]. Our study is among the first in which CGM metrics are compared in insulin or metformin groups in an RCT. Previous trials comparing metformin vs insulin in women with GDM include the MiG trial [2] and the MiG TOFU (metformin in gestational diabetes: the offspring follow-up) trial [11]. In Rowan et al.'s MiG trial comparing metformin versus insulin for the treatment of GDM to assess efficacy and safety, 751 women were included. The primary outcome of the trial was a composite of neonatal hypoglycemia, respiratory distress, need for phototherapy, birth trauma, five-minute APGAR score less than 7, or prematurity. Their trial demonstrated metformin alone or with supplemental insulin is not associated with increased perinatal complications as compared with insulin [2]. In the MiG TOFU trial, the primary objective was to compare body composition and metabolic outcomes at seven to nine years in the offspring of women with GDM randomized to metformin (±insulin) or insulin treatment during pregnancy. The study demonstrated that metformin or insulin for GDM was associated with similar offspring total and abdominal body fat percentage and metabolic measures at seven to nine years [11].

To date, there are no RCTs comparing either different CGM metrics or different targets for CGM metrics in pregnancies complicated by diabetes. In a prospective observational study of 162 women with GDM mean glucose was significantly higher in pregnancies complicated by large for gestational age (LGA) status. Neither TIR nor glycemic variability were associated with LGA status. Functional data analysis demonstrated that mean glucose was significantly higher overnight in pregnancies complicated by LGA status versus those without this complication. An RCT by Spaulonci and colleagues comparing metformin with insulin showed better blood glucose control in the metformin group [12]. There was no difference in maternal outcomes like pre-eclampsia, preterm labor, and cesarean section between both groups. A systematic review and meta-analysis of 24 studies comparing metformin and Insulin reported that metformin did not increase the incidence of preterm labor, pre-eclampsia, cesarean delivery, and small for gestational age babies [13]. Further, it was found that metformin lowered the risk of hypertension during pregnancy, LGA fetuses, macrosomia, and neonatal hypoglycemia [14].

Data from CGM devices in GDM is in the evolving phase. Our study result has added to the existing knowledge base. At this juncture, we are not giving a generalized statement from our study result. CGM metrics which we used in our study were based on 14 days of data but due to logistic constraints we have not compared neonatal outcomes. This gives an opportunity for future studies. The study's primary constraint lies in its limited sample size. Nonetheless, we achieved sufficient statistical power, and it would have been unethical to enroll additional participants. Despite the small dataset, we successfully identified that metformin is non-inferior to insulin for treating GDM in women.

## Conclusions

In the present study, the metformin and insulin groups didn’t differ in CGM metrics. Metformin, which is an oral drug that can be used in GDM, showed non-inferiority in comparison to insulin. This has clinical application in treating women with GDM where many patients who are not ready to take insulin because of injection fear, can be tried on metformin.
